# 

**Published:** 1888-06

**Authors:** 


Volume LVI.] JUNE, 1888. [Number 6.

### The CHICAGO MEDICAL JOURNAL AND EXAMINER

EDITOR:
S. J. JONES, M.D., LL.D

RAND, McNALLY & COMPANY, PRINTERS.

1888.

Entered at the Post Office at Chicago, for transmission through the mails as second-class matter.

